# Vaccination of Ukrainian Refugees: Need for Urgent Action^[Author-notes ciac276-FM1]^

**DOI:** 10.1093/cid/ciac276

**Published:** 2022-04-11

**Authors:** Piotr Rzymski, Halina Falfushynska, Andrzej Fal

**Affiliations:** Department of Environmental Medicine, Poznan University of Medical Sciences, Poznań, Poland; Department of Orthopedagogy and Physical Therapy, Ternopil V. Hnatiuk National Pedagogical University, Ternopil, Ukraine; Collegium Medicum, Warsaw Faculty of Medicine, Cardinal Stefan Wyszyński University, Warsaw, Poland

**Keywords:** war refugees, infectious diseases, pandemic, vaccine hesitancy, public health

## Abstract

The unprovoked aggression of Russian military forces on Ukraine in February 2022 has caused a high influx of refugees, including children, to neighboring countries, particularly Poland. This caused additional pressures on the healthcare system and the need to meet challenges for public health, such as those related to infectious diseases. Here, we discuss the potential epidemiological risks associated with the war-induced influx of refugees (coronavirus disease 2019, measles, pertussis, tetanus, and poliomyelitis) and highlight the need for their swift management through institutional support, educational campaigns, counteracting antiscience misinformation, and pursuing vaccinations of refugees but also improving or maintaining good levels of immunization in populations of countries welcoming them. These are necessary actions to avoid overlapping of war and infectious diseases and associated public health challenges.

## Teaser

The war in Ukraine in 2022 and the associated influx of refugees to neighboring countries is a call for urgent measures to mitigate risks related to infectious diseases, particularly coronavirus disease 2019, measles, pertussis, tetanus, and poliomyelitis, because of low vaccination rates.

## INTRODUCTION

The overlapping of war and infectious diseases is known to be a significant cause of human suffering and death. During military conflicts, the affected populations are displaced, with millions of refugees often forced to flee their territory, leading to overcrowding and limited access to food and clean water [1]. The countries receiving the refugees also face public health challenges because of increased demand for hospital and outpatient care and the need to adapt the healthcare system to refugees’ novel and specific requirements related to disease control programs, such as vaccination.

The unprovoked invasion of Russian military forces on independent Ukraine on 24 February 2022 has rapidly led to a humanitarian crisis. According to the United Nations’ refugee agency, 3 million people have been forced to flee Ukraine by mid-March 2022. The majority of them, 1.8 million (including 1 million children), have crossed the border with Poland [2]. Numerous refugees centers providing aid have been established across the country. Although some consider Poland only a transit point, a majority will choose to stay in this country, and additional millions may arrive as the war continues. The other countries experiencing a significant (but at least a few-fold lower than in Poland) refugee influx from Ukraine by mid-March 2022 included Romania, Hungary, Slovakia, and the Republic of Moldovia [2].

The military conflict and its multifaceted consequences have completely overwhelmed other challenges. However, the potential epidemiological risks related to the displacement of many Ukrainian inhabitants must be understood and mitigated. This is particularly relevant considering that the migration crisis coincides with the ongoing coronavirus disease 2019 (COVID-19) pandemic, whereas countries receiving the highest number of Ukrainian refugees (ie, Poland) are prone to antiscience and antivaccine sentiments, likely magnified by Russian influences [3, 4]. In turn, Ukraine was subject to healthcare misinformation tactics pursued by the Russian parties, particularly in conflict-affected areas, aiming to undermine trust in health authorities [4, 5]. Hence, this paper aims to discuss the potential 5 major vulnerabilities related to infectious diseases (ie, COVID-19, measles, pertussis, tetanus, and poliomyelitis) and ultimately offers recommendations for countries receiving a high number of refugees from Ukraine.

## COVID-19

In Ukraine, similar to many other countries, the COVID-19 pandemic has caused significant challenges to the healthcare system. Until the war, 5.04 million severe acute respiratory syndrome coronavirus 2 (SARS-CoV-2) infections have been identified (11.4% of the population), including 1.19 million in 2022. The death toll amounted to 112 459 (a 2.2% mortality rate), including 10 159 in 2022 before the war. The COVID-19 vaccination campaign in Ukraine started on 24 February 2021 [6], precisely 1 year before the Russian military invasion. Overall, 6 vaccines were authorized: messenger RNA vaccines, BNT162b2 (BioNTech/Pfizer, Germany/USA) and messenger RNA-1273 (Moderna, USA), modified adenoviral vector vaccines Ad26.CoV2.S (Janssen/Johnson&Johnson, Belgium/USA), AZD1222 (Oxford/AstraZeneca, UK/Sweden), and its Indian-made version Covishield (Serum Institute of India), as well as inactivated vaccine CoronaVac (Sinovac Biotech, China). At the beginning of 2021, the Ministry of Health in Ukraine authorized the use of booster dose for adults who completed an initial vaccination regime at least 6 months earlier. By 24 February 2022, the percentage of the population with a complete initial protocol reached only 35%, whereas only 1.7% of inhabitants received a booster dose [7]. At the same time, the average rate of initial COVID-19 vaccination in the European Economy Area reached 72%, whereas 51% of the population received a booster dose [8]. There are 4 main reasons behind such low vaccination rates in Ukraine: (1) delayed introduction of vaccines compared with developed regions; (2) significant shortages of doses supply; (3) an online influence operations campaigns, possibly a part of hybrid war, engaged in disrupting the national vaccination in Ukraine; and (4) widespread vaccine hesitancy also among healthcare workers who were the first to receive the vaccination [5, 9]. Moreover, as reported by the media, the demand for fake vaccination certificates was high, particularly after the European Union's decision to accept Ukrainian digital documents [10].

Considering all of these reasons, most Ukrainian refugees are unvaccinated. This group also comprises high-risk individuals, including the elderly, obese, and those with comorbidities. For obvious reasons, refugees crossing the Ukrainian-Polish border are exempt from the mandatory COVID-19 testing or quarantine [11]. The adherence to mask-wearing in humanitarian aid centers is low or non-existent. At the same time, the COVID-19 vaccination rate in the Polish population in March 2022 (59% completed the initial regimen, 30% received a booster dose) is below the European average [8]. The same applies to other countries experiencing a significant refugee influx from Ukraine (Table 1).

**Table 1.** The Coronavirus Disease 2019 Vaccination Rate in Countries Experiencing a Significant Refugee Influx From Ukraine During the First Three Weeks of the War in 2022

	Share of People With a Complete Initial Protocol by 15 March 2022, % [7]	Number of Refugees From Ukraine by 15 March 2022 [2]	Increase in Population Size Because of Refugee Influx, %
Poland	59	1 830 711	4.8
Romania	42	459 485	2.4
Republic of Moldovia	26	337 215	13.0
Hungary	64	267 570	2.7
Slovakia	51	213 000	3.9

Some percentage of refugees will stay only temporarily in the listed countries and travel further.

All in all, efforts must be undertaken to increase COVID-19 vaccination rates, both among Ukrainian refugees and the population of countries receiving their high number (ie, Poland and others) (Table 1). SARS-CoV-2 reveals a clear seasonality pattern in temperate zones, with the highest number of infections noted in the autumn-winter season [12]. During this period, the vaccination rate shows significant inverse correlations with the rate of COVID-19 hospitalizations, admissions to intensive care units, and deaths [13]. Besides making COVID-19 vaccines available for refugees (a step already undertaken in Poland [11]), additional vaccination promotion campaigns must be launched to ensure good vaccine coverage before the autumn-winter 2022 season. Such campaigns, aiming at refugees (prepared in the Ukrainian language) and the receiving population, should stress the pivotal need to decrease COVID-19-related pressure on the healthcare system when it faces significant challenges related to the inflow of refugee patients requiring hospitalization and outpatient care.

## MEASLES

Measles is a highly contagious viral disease that is vaccine preventable. Because of low vaccine coverage, particularly in some regions of Africa and Asia, the measles virus remains a cause of considerable morbidity and mortality, especially in children in resource-poor settings [14]. In 2019, approximately 873 000 measles cases (including 207 500 with fatal outcomes) were noted worldwide, the highest number reported since 1996 [15]. The main driver of this was insufficient vaccine coverage in some regions and a failure to vaccinate children on time with 2 doses of measles-containing vaccine.

**Figure 1.**
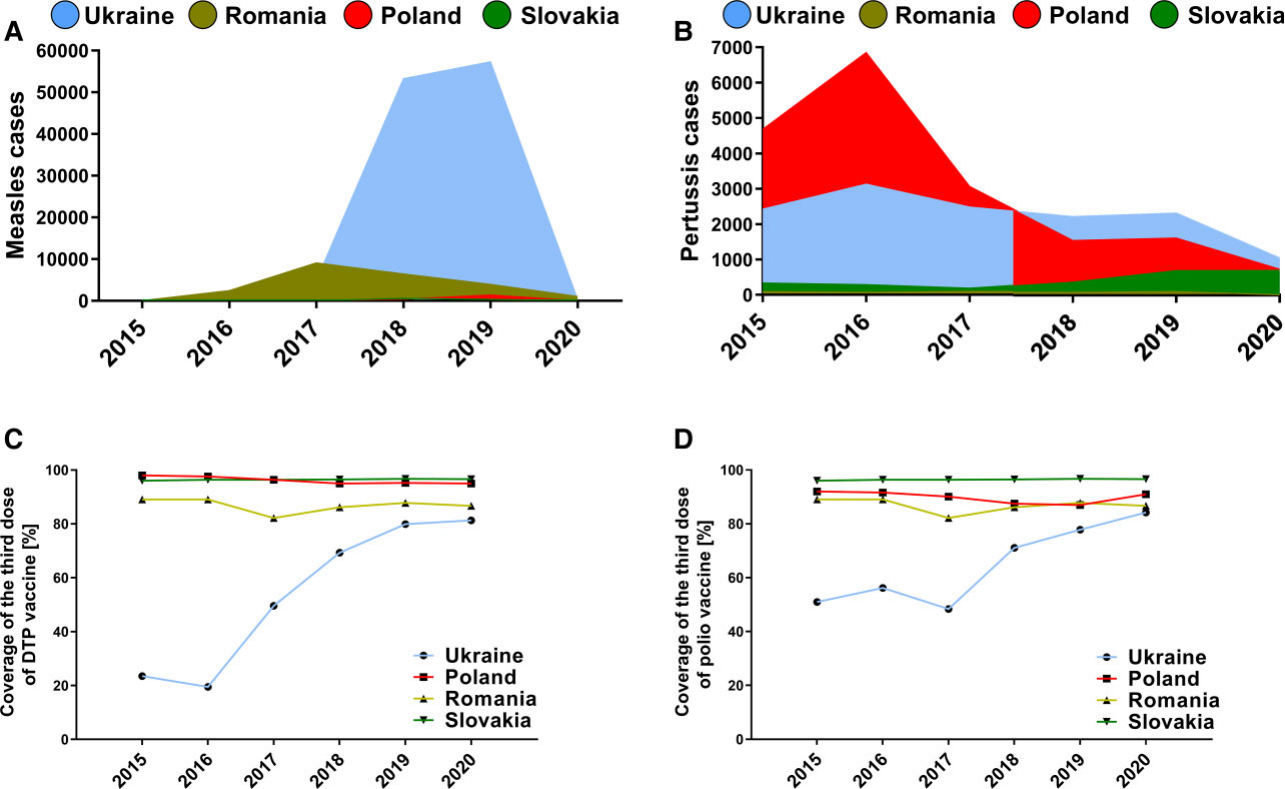
Measles (*A*) and pertussis (*B*) cases in Ukraine and selected countries receiving a high number of Ukrainian refugees and rates of administration of the third dose of pertussis vaccine (DTP) (*C*) and polio vaccine (*D*) in the eligible population. Based on World Health Organization data [15].

In Ukraine, the most recent measles outbreak started in 2017, during which the number of cases increased 47-fold compared with the preceding year (Figure 1*A*). In 2019, it exceeded 57 000. During the first decade of the 2000s, the coverage of the first and second doses of measles-containing vaccine (given as MMR vaccine against measles, mumps, and rubella) ranged from 94% to 99%. However, it had fallen to 56% and 41% in 2010, respectively, because of political conflicts, subsequent delays in supplying healthcare units with a sufficient number of vaccine doses, and increasing vaccine hesitancy. It remained low in 2015 and 2016 and led to an increase in measles cases in subsequent years (Table 2). However, from 2017, the vaccine administration started to increase because of an awareness-raising campaign on vaccinations undertaken by health authorities. In 2020, the total number of reported measles cases fell to just 264, which may be because of improved vaccination coverage. However, this needs to be interpreted with caution because the COVID-19 pandemic has affected the surveillance of diseases in various world regions [14]. As shown in Table 2, the measles vaccination coverage had decreased by approximately 10% in 2020, likely because of the COVID-19 pandemic and hospital reorganization to focus exclusively on emergency care. The MMR vaccination rate in Ukraine was below optimal levels despite being mandatory for children [16].

**Table 2.** The Coverage of Measles Vaccination in the Eligible Population of Ukraine and Selected Countries Receiving War Refugees in 2022

Country	Dose	2015	2016	2017	2018	2019	2020
Ukraine	First	56.0	43.0	85.5	91.0	93.2	84.9
	Second	57.0	31.0	83.5	89.5	91.7	81.9
Poland	First	96.0	95.5	94.0	92.9	92.0	80.3
	Second	94.0	93.4	93.0	92.4	93.0	95.3
Romania	First	85.7	85.8	86.5	89.6	89.5	87.3
	Second	80.0	76.3	74.7	80.9	75.8	75.1
Slovakia	First	95.2	95.2	95.8	96.1	96.2	96.0
	Second	97.6	97.4	97.4	97.4	97.8	97.9

Based on World Health Organization data [15].

Compared with the situation in Ukraine, the countries receiving war refugees had a lower number of measles cases in recent years. However, between 2015 and 2020, its mean annual number in Romania was 3800, whereas Poland noted a nearly 4-fold increase in identified cases between 2018 and 2019. At the same time, the uptake of the first dose of measles-containing vaccine in Poland revealed a decreasing trend between 2015 and 2020 (from 96.0% to 80.3%) and was far from optimal in Romania (<90%) (Table 2).

A high influx of children refugees from Ukraine, mainly to Poland, calls for urgent action to ensure high measles vaccine coverage. The measles vaccination is currently mandatory in Poland, Hungary and Slovakia [17], and this obligation should also include Ukrainian children. Simultaneously, education campaigns are needed to increase awareness of measles and its pathophysiology in refugees and the country's population. It is pivotal to adhere to the 2-dose guideline (the first dose at 12–15 months of age, the second dose at 4–6 years of age) by the World Health Organization (WHO) to ensure high seroconversion rates [14]. Measles virus is highly transmissible; therefore, vaccine coverage (with both doses of vaccine) must be maintained at the level of at least 95% of the eligible population. From countries included in Table 2, only Slovakia achieved it throughout 2015–2020. Particular efforts must, in turn, be undertaken in Poland, which received the highest number of Ukrainian refugee children.

## PERTUSSIS AND TETANUS

Pertussis is a highly contagious respiratory disease caused by Gram-negative bacterium *Bordetella pertussis*. Although it is clinically most significant in newborns and children, it can also occur in adults whose prolonged cough can be its only manifestation. Between 2000 and 2019, the mean annual number of cases reported globally to WHO was 167 000. Compared with this figure, identified infections in 2020 decreased by 59%, likely because of worse surveillance during the COVID-19 pandemic. Pertussis is largely undiagnosed and underreported (eg, 24 million new cases and 160 000 deaths were estimated to occur in 2014 alone, with the African region contributing the most significant proportions) [17]. In Europe, the incidence <1 case per 100 000 population was recommended by WHO to be achieved by 2000. However, this goal was not reached by 2020 [15].

The mean number of identified pertussis cases in Ukraine between 2015 and 2020 was 2268 per year, ranging from 3132 (2016) to 1041 (2020) [15]. The administration of the third dose of the diphtheria-tetanus toxoid and pertussis vaccine (DTP) vaccine was very low in children in 2015–2017 despite being mandatory according to a Decree of Health Ministry of Ukraine issued in 2014 [16]. However, it gradually increased to reach a coverage of 81% of the eligible population in 2020 (Figure 1*C*).

During the same period, the pertussis control in Poland and Slovakia was still far from optimal (Figure 1*B*). This is despite high vaccine coverage in these countries (Figure 1*C*). This can be explained by poor vaccine immunogenicity in some individuals, the emergence of new bacterial variants [18], and the waning of vaccine-induced immunity [19]. However, the primary goal of pertussis vaccination is to decrease clinical severity and disease burden. Dosing schedules differ between countries, although the primary immunization with 3 doses of the pertussis vaccine (given in the form of DTP) within the first 6 months of life is routinely practiced in the majority of regions, with selected countries recommending a booster dose in adolescence to counteract waning immunity [20]. In Poland, Slovakia, and Hungary, the administration of the DTP vaccine is mandatory, and is recommended in Romania [21]. Refugee children staying longer in these countries (> 3 months) should also be subject to compulsory vaccination; the decision should be accompanied by an educational campaign, similar to actions undertaken in the context of measles vaccination.

The other potentially relevant issue related to bacterial infectious diseases is the risk of tetanus. The tetanus toxoid is part of the DTP vaccine, in which administration in the eligible groups of children was generally low in the 2015–2020 period although this has gradually improved (Figure 1*C*). The seroepidemiological survey conducted in 2017 among Ukrainian children born 2006–2015 indicated the adequate levels of protection (≥80%) in areas of Kyiv, Odessa, and Sumy, from which a high number of refugees fled in the first weeks of the war. Some other regions, such as Zakarpattia, displayed a low level of protection (62%), highlighting that tetanus vaccine coverage varied across the country [22]. The level of protection in adults is unknown. Ukraine endorses WHO guideline to repeat vaccination against tetanus in adults (using DTP or tetanus and diphtheria TD vaccine) every 10 years to counteract the waning of humoral immunity. The percentage of adults applying to this recommendation in Ukraine is unknown, but it is likely to be extremely low because this is not a mandatory practice. Most refugees are not injured or wounded and thus not at high risk of contracting *Clostridium tetani*. However, countries receiving a high influx of refugees should consider securing extra doses of antitoxin to prevent tetanus in those who have wounds (eg, from escape-related accidents and injuries) and have not been fully vaccinated.

## POLIOMYELITIS

Poliomyelitis (polio) is an infectious disease caused by the poliovirus with a high range of severity, including meningitis and flaccid paralysis in the most clinical severe form. The motor neurons can be damaged throughout the illness, ultimately leading to irreversible muscular dysfunction. In some cases, death from respiratory dysfunction can occur. Poliomyelitis is a vaccine-preventable disease requiring multiple vaccine doses to ensure high effectiveness [23]. Typically, 3 doses are given in the following age intervals 2 months old, 4 months old, and 6–18 months old, followed by a booster dose in children 4–6 years old. Owing to global vaccination efforts ongoing for nearly 4 decades, poliomyelitis is now considered nearly eradicated, with poliovirus remaining endemic in Afghanistan and Pakistan; in 2020, these countries reported a total of 140 cases [24]. Since 2007, at least 80% of the eligible global population has received a third dose of polio vaccine [15].

In Ukraine, 3-dose polio vaccine coverage declined from 91% in 2008 to 15% by mid-2015 when 2 unrelated unvaccinated children in the Zakarpattia region were paralyzed by a highly divergent circulating vaccine-derived poliovirus type 1 (cVDPV1) [25]. The first Ukrainian case of poliomyelitis since these cases was identified in autumn 2021 in an unvaccinated 17-month-old girl and resulted in acute flaccid paralysis and was followed by identification of virus in her 7 siblings and 8 community contacts who did not display paralytic symptoms. This outbreak was linked to the cVDPV2 originating in Pakistan. Overall, 20 cases were identified in the Rivne and Zakarpattia regions until the end of 2021 [26].

Ukrainian children are more vulnerable to contracting poliovirus because of the lower vaccination rates than in other countries. This is despite the Decree of the Health Ministry of Ukraine issued in 2014 that enforced mandatory children vaccination against poliomyelitis [16]. In 2016 and 2017, only 56.2% and 48.4% of the eligible population received a third dose, respectively (Figure 1*D*). A national immunization campaign targeting nearly 140 000 Ukrainian children throughout Ukraine was scheduled to be launched in February 2022 but was forced to be stopped because of the Russian invasion [27]. Therefore, some refugee children are not vaccinated against poliomyelitis or did not finish their schedule. It is pivotal to ensure their vaccination. This goal should be achieved by raising awareness of poliomyelitis threats among refugees and including their children in the mandatory polio vaccination programs already enforced for children living in Poland and other countries receiving many refugees from Ukraine. These populations are currently well vaccinated to ensure a good level of protection; the herd immunity threshold for poliovirus is 80%–85% [28].

## CONCLUSIONS AND RECOMMENDATIONS

The war in Ukraine has led to the high influx of refugees to neighboring countries, particularly Poland. Most fleeing individuals are not vaccinated against COVID-19, whereas the vaccination against measles, poliomyelitis, and pertussis in refugee children may not be sufficient. Despite mandatory measles and pertussis vaccinations in Poland, a country receiving the highest number of refugees from Ukraine, the antivaccine movements are drawing some parents away from the decision to vaccinate their children. All of this can lead to significant epidemiological threats and challenges under mass migration and overcrowding. This does not only concern countries neighboring Ukraine because some refugees are traveling further, especially to other European regions, while vaccine-preventive diseases respect no borders.

The vaccination promotional campaigns aiming at refugees and populations of countries receiving them must be immediately enforced. This requires the involvement of local public health authorities by issuing vaccination plans for refugees and prioritizing the administration of MMR, DTP, and polio vaccines in refugee children. The vaccination status of each Ukrainian child entering the country should be established; in case of lack of documentation, they should be treated as unvaccinated. The promotional efforts must be supported by primary healthcare units and the medical community working with refugee parents and children. The informational materials (eg, flyers) prepared in nonspecialist Ukrainian language, explaining the immunological mechanism of vaccination and its benefits, counteracting most frequent myths, and presenting the most common side effects, should be prepared and distributed in refugee shelters, hospitals, primary healthcare units, and medical doctors’ offices (for example material, see [29]). The refugee children staying longer than 3 months in a particular country should be subject to immunization according to the local guideline on mandatory and recommended vaccination. In countries such as Poland, Slovakia, and Hungary, they should be subject to compulsory vaccination with MMR, DTP, and polio vaccine.

In the case of COVID-19 immunization, which is not required in Poland and other countries receiving a higher influx of refugees, promotional campaigns, particularly addressing adults from high-risk groups, should be launched. This requires preparation and wide distribution of informational materials explaining the benefits of COVID-19 vaccination on individual and public health levels (including their effectiveness against the recently emerged SARS-CoV-2 variants), types of COVID-19 vaccine approved in a particular country, their vaccination protocol, and mechanism of action, and steps to prepare for vaccination (for example, material see [30]). Refugees lacking valid documentation of vaccination should be treated as unvaccinated. The potential antivaccine misinformation aiming to destabilize public health in countries welcoming Ukrainian refugees should be expected, and efforts must be undertaken to counteract it by active surveillance of online social media and antivaccine movements by the national security agencies ceasing attempts of foreign powers to disintegrate public health. At the same time, economic and infrastructural resources must be mobilized to ensure undisturbed access to additional doses of vaccines and other preventive measures. The local health authorities should receive financial support from the European Union, but also actively cooperate with WHO and public-health partnerships such as GAVI to improve access to vaccines for Ukrainian refugees.

## Notes

***Author Contributions.*** P. R. conceived the idea and design for this manuscript; P. R., H. F., and A. F. wrote the first draft. All authors revised and approved the final draft.
